# Epigenetic developmental mechanisms underlying sex differences in cancer

**DOI:** 10.1172/JCI180071

**Published:** 2024-07-01

**Authors:** Joshua B. Rubin, Tamara Abou-Antoun, Joseph E. Ippolito, Lorida Llaci, Camryn T. Marquez, Jason P. Wong, Lihua Yang

**Affiliations:** 1 Department of Pediatrics,; 2 Department of Neuroscience,; 3 Department of Radiology,; 4 Department of Biochemistry and Molecular Biophysics,; 5 Deartment of Genetics Washington University School of Medicine, St. Louis, Missouri, USA.

## Abstract

Cancer risk is modulated by hereditary and somatic mutations, exposures, age, sex, and gender. The mechanisms by which sex and gender work alone and in combination with other cancer risk factors remain underexplored. In general, cancers that occur in both the male and female sexes occur more commonly in XY compared with XX individuals, regardless of genetic ancestry, geographic location, and age. Moreover, XY individuals are less frequently cured of their cancers, highlighting the need for a greater understanding of sex and gender effects in oncology. This will be necessary for optimal laboratory and clinical cancer investigations. To that end, we review the epigenetics of sexual differentiation and its effect on cancer hallmark pathways throughout life. Specifically, we will touch on how sex differences in metabolism, immunity, pluripotency, and tumor suppressor functions are patterned through the epigenetic effects of imprinting, sex chromosome complement, X inactivation, genes escaping X inactivation, sex hormones, and life history.

In humans, cancer occurs more commonly in males, and males die more rapidly of their disease compared with females (1). When we observe significant sex differences such as these, they are the result of sexual differentiation, a continuous process from gametogenesis to death. Sex differences in cancer incidence and outcome arise through multiple mechanisms. First are interactions between imprinting, chromosomal and hormonal sex, and developmental and life history effects on epigenetics. Together, these produce significant sex differences in growth regulation, metabolism, immunity, longevity, cell cycle regulation, response to DNA damage, p53 and retinoblastoma protein (pRB) function, among other determinants of health and lifespan (1). Second are the measurable effects of behaviors, exposures, and access to medical care, which account for variable proportions of overall cancer risk (2). These stressors and behaviors include those associated with gender expectations, roles, and identity. We will continue to refer to the differences we are discussing as sex differences, while recognizing that sex cannot be fully disentangled from gender, which also plays an important role in cancer risk.

Sex differences are individualized and vary in magnitude as a function of age. Thus, there is no complete dichotomy between being *male* or *female*. Despite this, it has proven productive to aggregate measured traits around male and female poles (3). While not complete or wholly accurate, categorical sex contains a lot of information about sex effects on the range of human phenotypes in health and disease. In this Review, we will use categorical terms based on sex chromosome complement to reveal a spectrum of sex-related phenotypes, while acknowledging their limitations.

Cancer risk is determined by interactions between inherited genetics, somatic mutations, epigenetics, exposures, and age. Familial and sporadic retinoblastoma (RB) proved the point. In 1971 Knudson made careful observations about RB and concluded that during a critical developmental window (<5 years of age), UV irradiation could cause tumors in anyone’s retina, but younger age at presentation, bilaterality, and multiplicity of tumors were all components of familial disease, while solitary tumors in one eye occurred sporadically in slightly older children (4). Regardless of germline RB status, no RBs occurred after 5 years of age. Thus, the window of risk for RB is narrow, and age is a context for its occurrence. RB incidence does not differ in males and females, but males are 3.4 times more likely to die of their disease (5). While the mechanisms underlying this and all the other significant sex differences in cancer incidence, treatment response, and survival remain to be fully defined, it is clear that sex, like age, is a critical contextual determinant of cancer risk. Here, we review some aspects of the epigenetics of sexual differentiation and consider how they factor in the genesis of cancer.

## The sex-biased epigenome always begins with the parents

**Figure 1:**
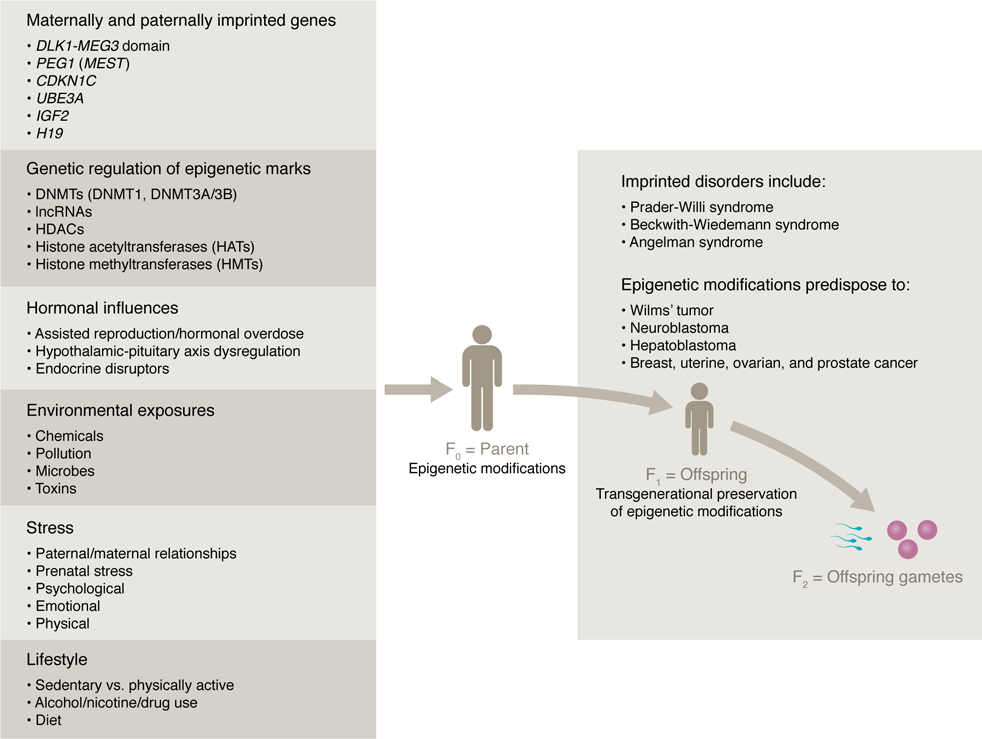
Mechanisms of transgenerational epigenetics. Transgenerational inheritance of epigenetic modifications are influenced by hormonal endocrine deregulators/disruptors and assisted fertility procedures and environmental exposures (chemicals, pollutants, toxins and pathogens), lifestyle factors (sedentary vs. physical activity, diet, alcohol, drug and nicotine use), maternal and paternal stressors (emotional, physical, psychological, and relationship dynamics) that can be passed on to subsequent generations: from parent (F_0_) to fetus (F_1_), to fetal gametes (F_2_), and so on. Such epigenetic modifications are known to alter the imprinting status of various genes (*DLK1-MEG3*, *PEG1/MEST*, *UBE3A*, *CDKN1C*, *IGF2*, *H19*) that manifest in imprinting disorders including: Prader-Willi Syndrome, and Angelman syndrome, and Beckwith-Wiedemann syndrome, among others. These syndromes are affiliated with cellular growth abnormalities predisposing the affected individual to an array of cancers including Wilms’ tumor, neuroblastoma, hepatoblastoma, and breast, uterine, ovarian and prostate cancers.

Genomic imprinting is the mechanism for transgenerational transmission of epigenetic adaptations to changing environmental conditions and the requirements for sex differences in reproductive success. Imprinting involves differential DNA methylation in the egg and sperm (Figure 1). Upon fertilization, these marks can be reprogramed in an XX- versus XY-biased manner, which then tailors the epigenome for future life as an XX or XY individual (6). The regulation of imprinted genes is governed by a complex interplay of DNA methylation, histone modifications, noncoding RNAs (microRNAs and long noncoding RNAs [lncRNAs]), and chromatin structure, with imprinted control regions (ICRs) playing pivotal roles in the establishment and preservation of imprinted marks (7).

During gametogenesis, specifically in primordial germ cells (PGCs), epigenetic marks are erased through global demethylation. This is followed by sex-specific DNA methylation patterns in sperm and eggs (8). Upon fertilization, the pronuclei of the egg and sperm merge, forming the zygote, which undergoes extensive epigenetic reprogramming of DNA demethylation and histone modification marks.

Transgenerational transfer of imprinted genes is maintained through multiple mechanisms, including ATP-dependent SWI/SNF and ISWI chromatin-remodeling complexes. These complexes play critical roles in maintaining the chromatin structure at imprinted loci (9). DNA methylation and histone modifications at imprinted loci are also preserved across generations of cells and individuals. In addition to DNA methyltransferase (DNMT) and histone-modifying enzyme activity, noncoding RNAs, which may be produced by the imprinted genes themselves, also participate in feedback loops and regulatory networks involved in maintaining imprinted genes (6, 7).

Imprinted genes play essential roles in embryonic growth, maternal-placental interactions, nutrient transfer, organogenesis, morphogenesis, and postnatal metabolism (8). The importance of imprinting is well illustrated by the pathological consequences of imprinting disorders (IDs). Anomalous DNA methylation patterns and loss of imprinting at specific genomic loci are associated with a range of developmental abnormalities and diseases, including Angelman syndrome (AS), Prader-Willi syndrome (PWS), Beckwith-Wiedemann syndrome (BWS) (7, 8), and Silver-Russell syndrome (6).

PWS and AS result in developmental and cognitive impairments that manifest along with multiple other syndrome-specific features. Both syndromes result from multiple mechanisms, including IDs involving chromosome 15q11–q13. Which syndrome occurs depends on whether there is loss of maternal expression of maternally expressed genes (MEGs) and *UBE3A* (in AS) or loss of paternal expression of paternally expressed genes (PEGs) (in PWS) (10). Sex differences in expression of MEGs and PEGs differs widely in a tissue-specific manner, with different sex-specific and shared tissues exhibiting either MEG- or PEG- dominant expression (11). It is important to note that sex differences in gene and protein expression are not required for sex differences in gene and protein activation and action (12–15). In murine models and human studies, the absence of sex differences in gene and protein expression was still associated with substantial differences in their action due to sex differences in chromatin accessibility, gene-regulatory networks, and intracellular signaling pathway regulation.

In BWS, the ID involves chromosome 11 and demethylation of the maternal *IGF2*, *LIT1*, KvDMR gene region (which regulates a cluster of genes) or methylation of the H19DMR region (also known as imprinting center 1 [IC1]) (16). IGF2 is an essential growth promoter in early fetal life, and H19DMR is an important negative regulator of its function. Normally, maternal *IGF2* is imprinted and silenced, while paternal H19 is imprinted and silenced. This antagonism between maternal and paternal imprints is essential for normal growth. In BWS, there is unopposed IGF2 function, resulting in an overgrowth syndrome with hemihypertrophy, hyperinsulinism, and a 10% increase in risk of childhood cancers such as multifocal bilateral Wilms’ tumor, hepatoblastomas, and neuroblastomas (17). In a rodent embryonic brain analysis, there was evidence for sex differences in IGF2 and H19 expression (18).

In addition to cancers that complicate BWS, variant ID methylation is associated with neuroblastoma (involving the DLK1-MEG3 imprinted domain) (6); acute myeloblastic leukemia (due to hypermethylation of the imprinted *NNAT* locus) (7); uterine leiomyoma (due to overexpression of PEG1 [also known as MEST]) (8); colorectal cancer (due to hypomethylation of H19 and *IGF2*, or IGF2 DMR0 hypomethylation) (7); breast cancer (due to PEG1 loss of imprinting) (6); and ovarian cancer (due to epigenetic alterations in the IGF2/H19 gene cluster or downregulation of *ARHI* and *PEG3*, whose products have tumor-suppressor function) (6, 7).

There are no reported robust or consistent sex differences in PWS phenotypes (19). There are, however, sex differences in the frequency of AS and PWS. These two syndromes can arise from nondisjunction during gametogenesis, resulting in uniparental disomy of pathogenic regions of chromosome 15. When this occurs during oogenesis, the offspring inherit two *maternally imprinted* copies of chromosome 15 and no paternally expressed copy of the gene and develop PWS. If the nondisjunction occurs during spermatogenesis, offspring inherit two *paternally imprinted* copies of chromosome 15 and develop AS. Because nondisjunction occurs more frequently during oogenesis than spermatogenesis, maternal uniparental disomy causing PWS is more common than paternal uniparental disomy causing AS (10). Thus, AS, PWS, and BWS all illustrate the presence of powerful sex differences in imprinting and the importance of balance between sex-adapted imprints for normal development, reproduction, disease risk, and long-term health.

In addition to supporting normal development, imprinting provides a mechanism for sex chromosome complement-adapted writing, erasing, and reading of DNA methylation marks for the transmission of positive and negative effects of the prior generations’ environmental stresses. Striking examples of sex differences in the transgenerational effects of stress are found in the metabolic reprogramming that has followed multiple famines, such as the Dutch famine of 1944–1945, the Great Chinese Famine of 1959–1961, as well as in Swedish famine cohorts (6, 7).

A number of sex differences in the Dutch famine effects have been identified. The first was a flip in the female-to-male birth ratio, from 47:53 before the famine to 52–51:48–49 in the affected cohort. Females exposed to famine in utero had higher rates of cardiovascular disease and cancer, with increased mortality from these causes than females born before the famine (20). Males exposed to in utero starvation had smaller intracranial volumes and on functional MRI (fMRI) studies appeared to have brains older than their chronological age, increased depression and anxiety, as well as inferior physical performance abilities (21). The children of individuals exposed to famine early in life also exhibit altered rates of obesity, hyperglycemia, type 2 diabetes, renal dysfunction/chronic kidney disease, and cardiovascular disorders (22). Interestingly, individuals in the Dutch famine cohort exhibit alterations in IGF2, but not H19, imprinting compared with unaffected siblings, underscoring the potential action of imprinting on transgenerational consequences of changing environmental stress (23). The Överkalix famine cohort and the Uppsala Birth Cohort Multigenerational Study (24–26) report similar sex differences in the transgenerational effects of famine.

We can expect highly personalized effects of environmental exposures and maternal/paternal stress on the programming and reprogramming of imprinted loci. Nutrition, lifestyle, stress, and exposure to chemicals and toxins impact the maintenance of imprinted alleles by affecting the activity of epigenetic regulators. Moreover, it is important to recognize that imprinting provides an established biological mechanism by which transgenerational gender stress can become ineluctably entangled with chromosomal and gonadal sex.

## Sex-biased DNA methylation changes across the lifespan

Methylomic sex differences are evident in gametes and persist throughout life (9). Interestingly, methyl marks change differently as a function of age in males and females (27). Sex bias in DNA methylation was powerfully demonstrated in a study of discordant twins. The methylome was shown to be less stable as a function of age in male twins compared with female twins (28). Loci with methylation changes in males were associated with longevity, multiple cancer-relevant pathways, and several cancers, specifically basal cell carcinoma, small cell lung cancer, melanoma, and glioma. Overall, male genomes may be undermethylated compared with female genomes — thus, more akin to hypomethylated cancer methylomes (29).

After formation of the testes, testosterone action suppresses DNMT activity. The effects of this can be seen in (i) the DNA hypomethylation that occurs in both sexes following perinatal testosterone exposure (30); (ii) in female littermates of male fetuses across species (31, 32); and (iii) human females with congenital adrenal hyperplasia (33) and human female twins of boys (34, 35). The developmental effects of testosterone exposure stably widen the sex differences in the methylome between male and female humans in utero (36, 37) and at puberty (38). In the premenopausal female rat, there is a reduction in DNA methylation rendering it more like the male hypothalamic methylome and decreasing epigenetic sex differences there (38, 39).

It is important to consider how sex differences in reprogramming and maintenance of imprinted loci, expression of DNMTs following fertilization, downstream regulation of gene expression, and age contextualize the genesis of cancer and the cancer methylome. To date, the glioblastoma (40), B cell chronic lymphocytic leukemia (41), and clear cell renal cell carcinoma (42) methylomes are reported to differ in male and female patients. In glioblastoma multiforme (GBM), promoter methylation and silencing of methyl-glutamyl methyltransferase (MGMT), a critically important resistance mechanism to standard-of-care temozolomide (TMZ) chemotherapy, occurs more frequently in female compared with male patients (43). This may directly relate to the superior radiographic response to radiation and TMZ observed in female patients with GBM (14). We expect that there will be additional reports of sex differences in cancer methylomes supporting sex-biased cellular and systems-level adaptations to oncogenic and treatment stressors.

## X chromosome inactivation and the biology of X-escapees

In the absence of X chromosomal aneuploidies (~1 in 1,400 births) (44), X chromosome inactivation (XCI) is a uniquely epigenetic female cellular event that achieves allelic balance for pseudoautosomal regions of X and Y chromosomes and endows female cells with differing capabilities compared with male cells. These sex differences arise through the downstream effects of genes escaping X inactivation (X-escapees) (45) and unbalanced gene expression on metabolism (*OGT* [encoding O-linked *N*-acetylglucosamine]; see below); immunity (*TLR7*) (46); lysine demethylase 6A (*KDM6A* or *UTX*) (47); and tumor protein 53 (p53), which binds to sequences in the X chromosome inactivation center (XIC) that are required for XCI and thus essential for maintaining the differentiated state (48). It will be important to determine how much the loss of XIC function, which occurs secondary to loss of canonical p53 function, contributes to malignant transformation and cancer progression. Incomplete X inactivation also affects genome-wide epigenetic regulation of gene expression. Lysine demethylase 5C (*KDM5C*), *KDM6A*, and ATRX chromatin remodeler (*ATRX*) are X-escapees that directly regulate epigenetics and play substantial roles in cancer protection. KDM5C functions as a histone 3 (H3) lysine 4 (K4) trimethylation (me3) (H3K4me3) demethylase. It is mutated in association with breast cancer, clear cell renal cell carcinoma (49), head and neck squamous cell carcinoma (SCC) (50), and acute myeloid leukemia (AML) (51). KDM6A is the primary H3 lysine 27 (K27) me3 (H3K27me3) demethylase. Biallelic expression appears to be protective against cancer. This has been most extensively explored in bladder cancer (52). ATRX is responsible for the genomic insertion of histone H3.3 and for the inhibition of the alternate lengthening of telomeres (ALT). Its mutation in XY cells results in ALT activation, thus contributing broadly to cancer development (53). Together, these genes provide protection against cancer by buffering against monoallelic loss of function and by their greater expression and activity in XX versus XY cells.

The absence of recombination between Y and X has allowed for the development of important differences in function between some Y and X paralogs, such as the oncogenic functions of testis-specific protein Y–encoded (TSPY) versus the tumor suppressor function of its paralog, testis-specific protein X–encoded (TSPX) (54) or the differences in demethylase activity between KDM6A and its paralog, UTY (55).

Activation of both X chromosomes is correlated with pluripotency, and XCI is required for differentiation (56). Variable levels of X are required for induced pluripotency (57). In rodents, complete reactivation is required, while in humans, it appears that induced pluripotency requires only partial reactivation of the silent X (58). The reacquisition of a dedifferentiated or pluripotent state is a feature of cancer stem cells. Thus, the presence of a second X chromosome provides multiple mechanisms of cancer protection or tumor suppression, including a buffer against heterozygous mutation of X alleles, the biology of X-escapees, and a barrier against the emergence of cancer stem cells. The tumor-suppressor effects of a second X chromosome are further supported by the markedly increased cancer risk in individuals with Turner syndrome (XO; ref. 59) and the decreased solid tumor risk in individuals with Klinefelter syndrome (XXY; ref. 60).

In addition to X chromosome dynamics in cancer, recently the role of loss of the Y chromosome (LOY), a frequent event in male aging (61), has been documented in a large number of primary male tumors, where in some cases it appears to be a driver event (62).

## Sex differences in an epigenetics-metabolism cycle

**Figure 2:**
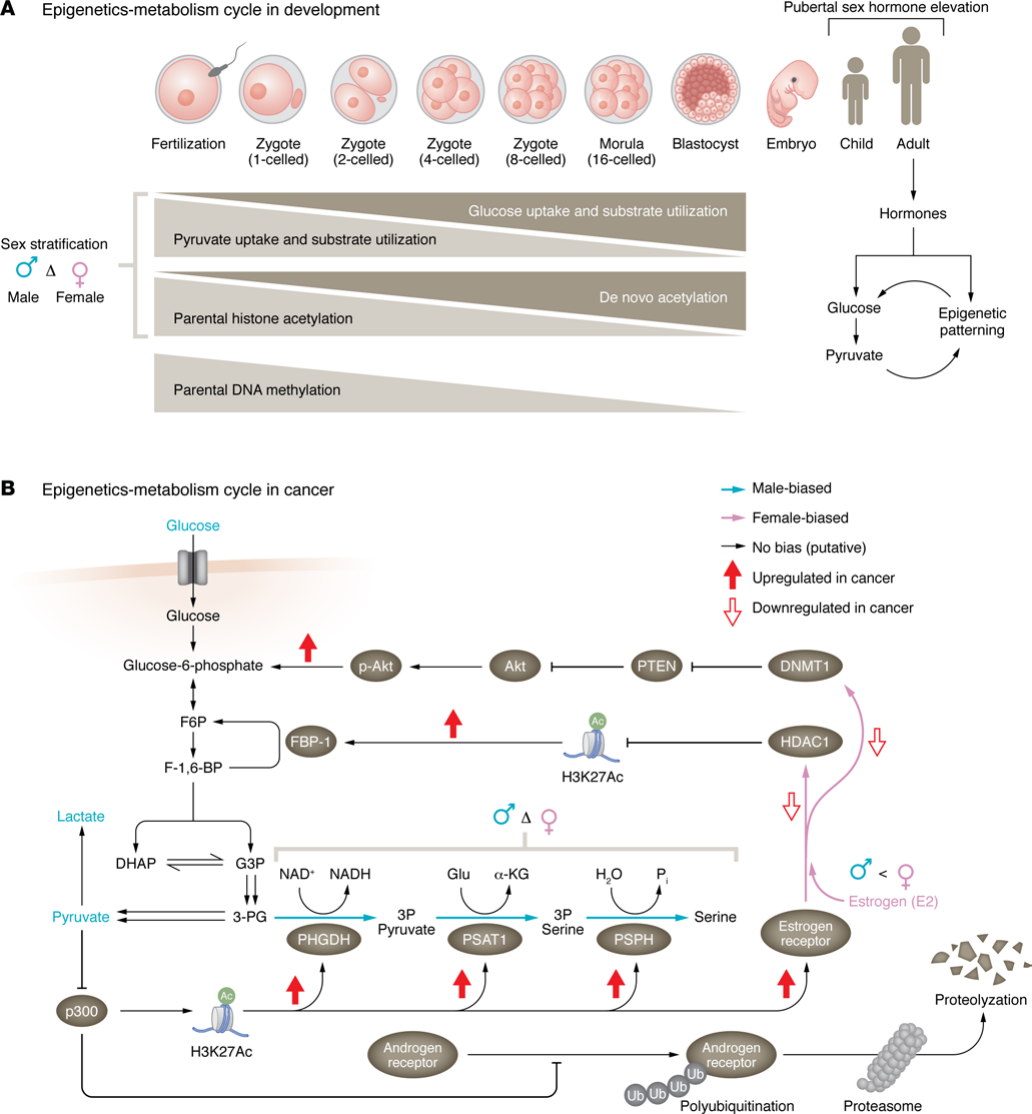
The epigenetics-metabolism cycle throughout development and cancer. (**A**) After fertilization, sex-adapted mammalian development and patterns of concomitant metabolic and epigenetic changes emerge. Early zygotic development is characterized by paternal epigenetic patterning and a pyruvate-fueled metabolism. The development from single-celled zygote to preimplantation blastocyst follows a progression from epigenetic erasure of parental epigenomes to sex-stratified de novo acetylation and establishment of a sex-stratified, glucose-centered metabolism. Postpubertal sex hormones further modulate metabolic and epigenetic feedback. (**B**) Following import into cells, glucose is phosphorylated to glucose-6-phosphate, isomerized to fructose-6-phosphate (F6P), further phosphorylated to fructose-1,6-bisphosphate (F-1,6-BP), and split into trioses dihydroxyacetone phosphate (DHAP) and glyceraldehyde-3-phosphate (G3P), before conversion to 3-phosphoglycerate (3-PG). 3-PG is subject to dual fates: glycolytic conversion to pyruvate and lactate or conversion to serine via the serine biosynthesis pathway. Additionally, pyruvate inhibits H3K27 acetylation (H3K27Ac) by targeting histone acetyltransferase (p300). Metabolites and arrows in blue indicate male-specific importance in cancers. In the context of cancer, p300-mediated H3K27 acetylation and androgen receptor acetylation promote upregulation of serine biosynthesis and estrogen receptor proteins and prevent polyubiquitination-mediated degradation of androgen receptor, respectively. Activation of estrogen receptor by estradiol (E2) results in transcriptional attenuation of histone deacetylase 1 (HDAC1) and DNA methyltransferase 1 (DNMT1). Decreased transcription of HDAC1 and DNMT1 promotes greater glucose consumption via downregulation of fructose bisphosphatase-1 (FBP-1) and phosphorylation of Akt, respectively. α-KG, α-ketoglutarate; PHDGH, phosphoglycerate dehydrogenase; PSAT1, phosphoserine aminotransferase 1; PSPH, phosphoserine phosphatase.

Mammalian male and female metabolic sex differences are dynamically shaped by and emerge from a combination of developmental, hormonal, and epigenetic mechanisms pruned by sexual selection (Figure 2). Unlike that of mammalian males, female physiology requires the judicious allocation of metabolic resources to potentiate dual support of maternal and fetal, as well as breastfeeding newborn, energetic needs. Therefore, male and female developmental programs diverge in service of the biological imperative to develop, reproduce, and yield healthy offspring from the moment of fertilization. Across all trimesters (63, 64) gestating male embryos are larger and more rapidly proliferative (65, 66) and exhibit higher energy demand (67–69) relative to female embryos. Early developmental literature has identified that male and female preimplantation blastocysts meet energetic demand by differentially prioritizing glucose (68, 70–75), amino acid (76), and lipid metabolism (77, 78). Experiments examining the effect of sequential X chromosome addition, Y chromosome deletion, and gonadal sex on metabolic substrate utilization confirm that prenatal sex biases are driven by sex chromosome complement during mammalian development (75, 79–83). However, the role of sex hormones in further modulating substrate utilization is also evident.

Sex hormones shape embryonic and adult mammalian epigenomes by recruiting DNA and histone-modifying enzymes to their substrates upon hormone receptor activation (84–87). In addition, sex hormones have been shown to modulate glucose, amino acid, and lipid metabolism across multiple healthy adult tissues. Androgen deprivation (88), attenuated production of estrogen or estrogen receptor (89), and menopause (90) strongly associate with adiposity, diabetes, cachexia (91–94), and cancer (95). Therefore, sex chromosome complement imparts a male or female metabolism that is modulated at the metabolic and epigenetic levels by the activating effects of sex hormones. However, epigenetic modification of histones and DNA requires cofactors produced by glucose (96), amino acid (97), and lipid catabolism (98). Because of this, changes in the metabolome and epigenome form a cycle that is sexually divergent from early embryogenesis and is postpubertally modified. Though sex differences in the metabolism-epigenetics cycle are highly underexplored, literature suggests these are of relevance to cancer.

Sex, sex hormones, and sex hormone receptor status may regulate the epigenome and metabolome of cancers. In SCC, estrogen receptor promoter hypermethylation is associated with worse prognosis and occurs more frequently in males regardless of their smoking status (99, 100). Treatment of male SCC cells with 17β-estradiol was observed to reverse promoter hypermethylation of the DNA repair gene *MGMT* (99, 100), which correlated with decreased expression of the epigenome-modifying enzymes DNMT1 and HDAC1. In other cancers, DNMT1 and HDAC1 activity modulates glucose metabolism. Elevated expression of DNMT1 supports greater glucose consumption in nasopharyngeal carcinomas (101). In hepatocellular carcinomas (102), HDAC1 attenuates gluconeogenesis via deacetylation of histone H3K27 at the enhancer region of *FBP1*, encoding fructose-1,6-bisphosphatase 1. These connections, if confirmed in SCC models, would suggest that male SCC patients may uniquely benefit from HDAC inhibition (HDACi). Male-specific benefits in response to HDACi have been identified in the context of developmental arsenic exposure (103) and Alzheimer disease (104). The connections between histone modification, substrate utilization, sexual differentiation, and cancer metabolism are developmentally rooted.

The shared metabolic needs of growing mammalian embryos and cancer cells are satisfied by similar mechanisms. The epigenetic erasure of imprinted methylation marks during embryogenesis precedes changes in glycolytic substrate utilization from pyruvate prior to compaction to glucose in morulae (105). The timed orchestration of these events suggests that de novo epigenetic patterning is exquisitely sensitive to changes in substrate utilization. Indeed, cancer cells and in vitro fertilized bovine embryos exhibit dynamic changes in histone acetylation patterns in response to glucose (106) and pyruvate (107) administration, respectively. Recently, histone acetylase p300 has been identified as a member of the glycolytic targetome (108). Administration of pyruvate to HCT116 human colon cancer cells significantly decreased global p300-mediated H3K27 acetylation (108). In breast (109) and prostate cancer models (110, 111), p300-mediated H3K27 and receptor acetylation determines estrogen receptor expression and androgen receptor (AR) stability, respectively. Developmentally, p300-mediated H3K27 acetylation functions to activate sex-determining region Y (*SRY*) gene expression and testes development (112). Inhibition of p300 in murine XY embryos leads to sex reversal (113). Therefore, the developmental substrate switch from pyruvate to glucose, which follows epigenetic reprogramming of imprinted loci, may potentiate p300-mediated male sexual differentiation. In the context of cancer, this same mediator promotes a permissive transcriptome to promote cellular proliferation (114). Sex differences in glucose uptake and flux of glucose into de novo serine biosynthesis have been reported in glioblastoma (115) and lung cancer models (116), respectively. Expression of de novo serine biosynthesis enzymes is controlled by the ATF4/ATF3 axis. Interestingly, ATF3-mediated upregulation of de novo serine biosynthesis transcripts requires recruitment of p300 to the serine biosynthesis gene loci in prostate, colon, and sarcoma cells (117). Currently, there remains a paucity of research exploring dynamic changes in the epigenome in response to amino acid, lipid, and carbohydrate substrate supplementation. These data highlight how such studies can improve our understanding of the epigenetic-metabolism cycle from both a developmental and cancerous context.

## Sex differences in the epigenetics of inflammation

Sex differences in immunity and inflammation exist throughout life, predisposing males and females to differing common disease phenotypes (118). Females exhibit stronger immune responses in general, resulting in greater vaccine responses, pathogen clearance, and a predisposition for decreased cancer incidence, but an associated predisposition for autoimmune disorders. Epigenetic programming in immune cells partially regulates these sex differences in immune response. A critical repressive mark differentially regulated in male versus female cells is H3K27me3 (119–121). The primary demethylase of H3K27 is the X escapee KDM6A (122). Higher KDM6A expression in female NK (119) and T cells (120) increased survival in mice with cytomegalovirus infection and glioblastoma, respectively. Moreover, sex differences in DNA methylation in monocytes, B cells, and T cells (123) and open chromosome accessibility in macrophages (124) may regulate sex differences in the effector functions of these immune cells.

The sex hormone profiles at different stages of life exert an immunomodulatory role (125) that can affect anticancer immunity. They do this in part through hormonal regulation of epigenetics. Both the onset of puberty (126, 127) and gender-affirming hormone therapy (128) induce changes in DNA methylation that are measurable in the blood. Further, DNA methylation is reduced in the hypothalamus of female rats upon transition to perimenopause (39). Some of these effects are due to differing levels of sex hormone receptors in immune cells (129). Expression of the AR on CD8^+^ T cells is known to promote T cell exhaustion in colorectal cancer (130), cutaneous melanoma (130), and prostate cancer (131). In CD8 T cells, AR activity and function is required to maintain sex differences in chromatin accessibility at regulatory transcription factor binding sites, which determine regulation of T cell exhaustion (130).

In contrast, estrogen promoted CD8+ T cell exhaustion in melanoma by a different mechanism. Estrogen decreased the ratio of M1 to M2 tumor-associated macrophages (TAMs), thereby creating an immunosuppressive tumor microenvironment (TME) (132). Similarly, in a mouse colon adenocarcinoma model, estrogen drove an immunosuppressive TME in the liver by way of myeloid-derived suppressor cells, which inhibited CD8^+^ T cell activation and promoted liver metastases (133). These studies suggest that even when CD8^+^ T cell exhaustion is similar in male and female tumors, the mechanisms underlying the exhaustion can differ.

Several imprinted lncRNAs have also been implicated in immune regulation and cellular senescence. These lncRNAs can modulate gene expression, chromatin structure, and signaling pathways involved in immune responses and aging-related processes. For instance, the paternally imprinted and silenced gene H19 has important roles in immune cell differentiation, cytokine production, and regulation of inflammatory pathways. Dysregulation of H19 expression has been associated with autoimmune diseases and inflammatory disorders (134). Loss of another imprinted lncRNA, MEG3, has tumor suppressor functions (135). The lncRNA *XIST*, which drives XCI, has been linked to female-biased autoimmunity and immune responses by regulating different immune cell populations (136–139). In female mouse macrophages and human monocytes, *XIST* expression is important for attenuating acute inflammatory responses (138). Dysregulation of *XIST* in both naive B and T cells promoted autoimmunity due to loss of proper maintenance in systemic lupus erythematosus (SLE) and primary biliary cholangitis (PBC), respectively (137, 139). Knockdown of *XIST* resulted in the differentiation of naive B cells into CD11c^+^ atypical B cells (139), while loss of XIST in naive CD4^+^ T cells from patients with PBC inhibited Th1 and Th17 differentiation (137). Interestingly, *Xist* expression in male transgenic mice resulted in a transcriptional shift in splenic CD4^+^ T cells and B cells to a more female-like state (136). The authors also show that in an SLE mouse model, diseased mice transgenic for *Xist* develop autoantibodies against the *Xist* ribonucleoprotein complex in a manner similar to that in WT female mice. These studies suggest that *XIST* is important for maintaining proper function of immune cells and that dysregulation of *XIST* can promote development of autoimmunity in a female-biased manner.

Besides cell-intrinsic regulation of immune cells in cancer, the efficacy of cancer therapies can be affected by the acellular and cellular TME (140, 141). Stromal, immune, and tumor cells secrete growth factors, cytokines, metabolites, and other signaling factors that can directly promote cancer progression by stimulating tumor cell proliferation, survival, and invasion and also indirectly promote cancer progression by regulating angiogenesis, the biophysics of tumor tissue, and immune function (142).

Immune activity in the TME can vary between nearly quiescent and inflammatory (141, 142). Senescent tumor and non-tumor cells are important determinants of the TME inflammatory state. Stable cell cycle arrest through senescence is continually induced in tumor tissue through oncogenic and replicative stress in tumor cells, oxidative stress in tumor and nontumor cells, and the DNA-damaging effects of radiation and chemotherapy (140, 143). Senescent cells regulate inflammation and tumor cell biology through the secretion of an inflammatory repertoire of growth factors, cytokines, and other factors known together as the senescence-associated secretory phenotype (SASP) (140). The SASP is a central paracrine regulator of non-senescent cell activity and function throughout the tumor tissue (141, 142).

Transition to a senescent state requires major shifts in cell state and predictably involves epigenetic reprogramming. Multiple studies demonstrate that senescence and the SASP involve changes to H3K27me3 status (144, 145). The primary methylator of H3K27 is polycomb repressive complex 2 (PRC2), a methyltransferase composed of multiple subunits, including embryonic ectoderm development (EED) and enhancer of zeste homolog 2 (EZH2) (140). Inhibiting PRC2 proteins induces the SASP. Inhibition of EED increased SASP expression in rhabdoid tumor cells. Likewise, downregulating EZH2 in human diploid fibroblasts (144) and melanoma cells (146) induced premature senescence, while its expression prevented Ras- and etoposide-induced senescence in human diploid fibroblasts (144). Further, inhibition of EZH2 in pancreatic ductal adenocarcinoma (141), small cell lung cancer (147), and cancer-associated fibroblasts (148) upregulated the SASP without changing the numbers of senescent cells.

**Figure 3:**
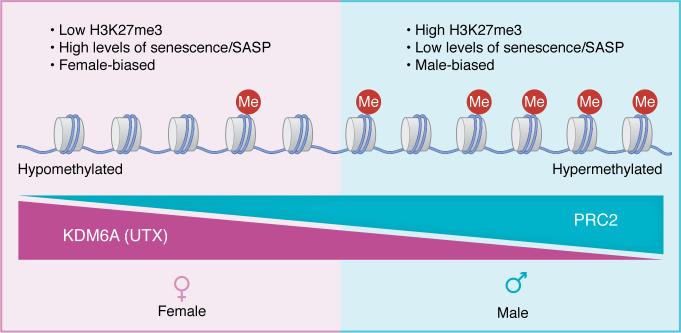
Sex differences in senescence may be due to sex-biased regulation of H3K27me3 levels. Female genomes tend to be hypomethylated on H3K27 at senescence-related genes due to increased expression of KDM6A, a demethylase that is an X-escapee, while male genomes are more likely to be hypermethylated on H3K27 due to male-biased effects of PRC2 and its subunits EED and EZH2. This results in a propensity of female cells to have higher levels of senescence and SASP compared with male cells.

Demethylases also regulate senescence. Overexpression of Jumonji domain–containing protein 3 (JMJD3), a KDM6 demethylase, induced senescence in glioma (149) and 293T cells (150). Likewise, overexpression of KDM6A induced senescence in 293T cells (150). In senescent mouse embryo fibroblasts (145) and mouse neurofibroma Schwann cells (151), induction of JMJD3 activated the *Ink4A* promoter, encoding p16, a marker of senescence. Together, these observations indicate that loss of H3K27me3 mediated by inhibition of PRC2 or upregulation of lysine demethylases promotes senescence and the SASP (Figure 3). As male and female cells exhibit different thresholds for senescence in different tissues and cancers (12, 142), there is the possibility that sex differences in H3K27 methylation could underlie the sex differences in senescence induction and tumor-promoting effects of the SASP.

Female tumor cells tend to have lower levels of H3K27me3 than male tumor cells, undergo senescence more readily, and express higher levels of SASP. What remains to be demonstrated is whether male and female cells are cleared equally well and quickly by the immune system. It will be the balance between senescence, SASP production, and senescent cell clearance that determines whether sex differences in senescence lead to sex differences in treatment response and tumor progression.

An emerging concept in novel cancer therapy is to leverage radiation and/or chemotherapy-induced senescence to arrest cancer cell division and then treat with senolytic agents to block the tumor-promoting effects of the SASP (143). Precision approaches targeting senescence and the SASP will require addressing the sex differences in senescence, its effect on tumor cell biology, and the associated immune responses, as well as the underlying epigenetics regulating both.

## Sex and cancer epigenetics

As described above, sex differences in epigenetics can affect cancer risk and outcome by regulating metabolism and immunity. There are also direct effects of sex differences in epigenetics on cancer cell biology. EZH2 is frequently upregulated in cancer, and high EZH2 expression is correlated with aggressiveness and a worse prognosis (152, 153). In non-small cell lung cancer (NSCLC), EZH2 expression is higher in males compared with females (152). Furthermore, knockout of both EZH2 and its related family member EZH1 increased expression of female-biased genes in male mouse livers, suggesting a shift to a more female-like state (154).

**Figure 4:**
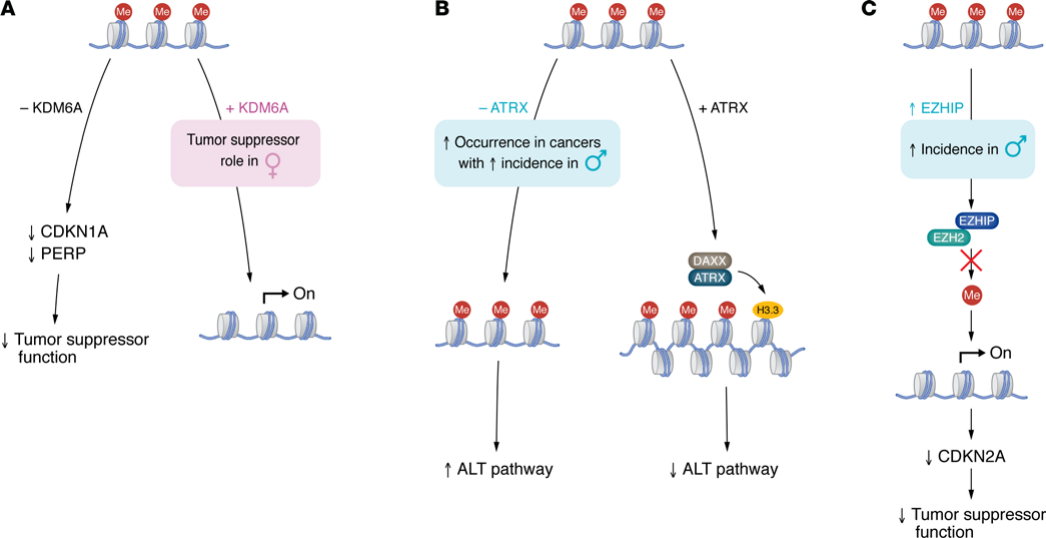
KDM6A and alterations in ATRX and EZHIP are associated with male-skewed cancers. (**A**) *KDM6A* (*UTX*) is a histone H3 lysine 27 di- and trimethylation (H3K27me2/3) demethylase that can lead to gene expression and serves as a tumor suppressor in bladder cancer in females. However, in mice, when *Kdm6a* is not present, downstream targets of the tumor suppressor p53, such as *Cdnk1a* and *Perp*, are not expressed. (**B**) ATRX interacts with DAXX and deposits H3.3 histone marks that cause chromatin compaction and inhibition of the ALT pathway. ATRX is mutated in some cancers including glioblastoma and oligodendroglioma that occur more often in males, and this leads to an upregulation of the ALT pathway in tumor cells, which can then cause tumor progression. (**C**) EZHIP interacts with the EZH2 subunit of PRC2 through its active site, causing loss of H3K27me3 levels, which can lead to gene expression. However, increased levels of H3K27me3 are observed at the *CDKN2A* locus in PFA ependymomas expressing EZHIP that suppress *CDKN2A* expression, thus lowering tumor suppressor function.

The X-escapee KDM6A opposes EZH2 function and can act as a tumor suppressor (Figure 4A) (155). In a murine model of bladder cancer, which occurs in 4 times as many males as females (156), knockout of *Kdm6a* decreased survival in female, but not male mice (121). Together, the EZH2 and KDM6A reports raise the possibility that H3K27 demethylation may underlie a female-biased tumor suppressor phenotype, while EZH2 and PRC2 activity may underlie a male-biased oncogenic phenotype (Figure 3). Interestingly, although it is a demethylase, *Kdm6a* exerts its tumor suppressive function in bladder cancer through both demethylase-dependent and -independent mechanisms by regulating the targets of the tumor suppressor p53, *Cdnk1a* and *Perp*, respectively (121).

A second X-escapee that exhibits sex-biased effects on cancer is ATRX (Figure 4B). ATRX interacts with the death domain–associated protein (DAXX) to deposit the histone variant H3.3 at repetitive regions in the genome, such as telomeres, to maintain a heterochromatic state (157). In order to maintain their telomeres and immortality, 15% of cancers utilize a telomerase-independent mechanism, the ALT pathway, and ATRX is important in suppressing this pathway (53). *ATRX* mutations are more commonly present in male cancer overall (158) and in a number of cancers with sex differences in incidence and outcomes, including, glioblastoma, oligodendroglioma (159, 160), gastric cancer (161), and nonfunctional neuroendocrine tumors of the pancreas and other sites (162, 163), suggesting that biallelic *ATRX* expression may endow female cells with cancer protection.

Loss of ATRX is accompanied by H3.3 mutation in diffuse midline glioma (DMG), a predominantly pediatric form of malignant glioma with extremely poor prognosis. The *K27M* mutations associated with DMG occur in *HIST1H3B* and *H3F3A*, the genes encoding histones H3.1 and H3.3 (164). H3K27M expression results in a global decrease in H3K27me3, aberrant regulation of gene expression, and abnormal neural differentiation (165), which may be related to its brain tumor-promoting effects.

Global loss of repressive H3K27me3 marks also occur in posterior fossa ependymoma type A (PFA), but through an alternate mechanism involving overexpression of Cxorf67, also known as EZH2-inhibitory protein (EZHIP) (Figure 4C) (166). EZHIP makes direct contact with the active site of the EZH2 subunit of PRC2 and inhibits its methyltransferase activity, resulting in loss of H3K27me3 marks (167). PFA ependymomas are 1.5 times more common in young males than young females (168), suggesting that loss of EZH2 function may more efficiently transform male compared with female PFA progenitor cells. Interestingly, while there is global loss of H3K27me3-repressive marks in EZHIP-overexpressing tumors, this is accompanied by increased levels of H3K27me3 at the *CDKN2A* locus in PFA ependymomas expressing EZHIP. This suppresses the expression of this critical tumor suppressor. Thus, like in *ATRX*, *KDM6A*, and *H3K27M*, *EZHIP* alterations are associated with male-skewed cancers. It will be important to determine whether these sex differences in incidence reflect different degrees of tumor protection in males versus females. Beyond sex differences in expression of epigenetic writers, erasers, and readers are sex differences in Brd4-bound enhancer usage that are not dependent upon differences in Brd4 expression, as they reflect sex differences in chromatin accessibility, much of which is patterned through in utero sexual differentiation (13).

In contrast to the male skew in cancers associated with loss of H3K27me3-repressive marks, loss of mixed-lineage leukemia 1 (MLL1 or KMT2A) expression through chromosomal translocation results in loss of the activating mark H3K4me3 in acute lymphoblastic leukemia (ALL) (169). MLL1 translocation–positive ALL occurs in 35% more females compared with males (170). This suggests that loss of this activating mark may more efficiently transform female ALL progenitor cells.

## Conclusion

All cancer-causing events at the cellular and systems levels must interact with the important nonrandom biological determinants of risk for cellular transformation and cancer progression. The changes that occur with development and aging in the genome and epigenome, across all biological scales, impose differing thresholds on cellular transformation and cancer progression. Sex also determines transformation thresholds and cancer progression, particularly metastatic disease, which is more frequent in male cancer cases of differing cancer types (171). Sex does this most powerfully through the genome-wide epigenetic consequences of imprinting and XCI on development and aging. Males and females develop and age differently. Among the associated features of sex-biased biology are the differing risks for cancer and cancer-related death. It is not possible to fully understand cancer biology and develop the most effective and least-toxic treatments for patients without comparing the sex differences in transformation and response to treatment that the epidemiology and biological data irrefutably demonstrate is there. Moving forward, the biology of sex differences in cancer must be seriously considered in research and drug development.

## Version 1 07/01/2024

Electronic publication
